# Guy's Hospital "Scandals" and the Southwark Guardians

**Published:** 1911-12-02

**Authors:** E. C. Perry


### GUY'S HOSPITAL "SCANDALS" AND THE SOUTHWARK GUARDIANS.

We are now able to publish the following letter, dated November 17, from Sir Cooper Perry, the Superintendent of Guy's Hospital, addressed by order of the House Committee to a subscriber to Guy's Hospital, who desired to know the facts. We refer to this matter on page 221 of the current issue.

Dear Sir,—Your letter with regard to Mrs. Beeston was read to the House Committee at their meeting yesterday, and I am directed to say that they thank you for your inquiries into her case.

You ask whether the report in the "Daily Express" is "correct"; and is it a fact that there was not a single empty bed in the Hospital on the 5th for a female erysipelas case, and consequently the house-surgeon had to refer the patient to the Poor Law authorities, who are under a legal obligation to afford medical relief to those whose circumstances render them eligible for such assistance.

On the day in question our ward for cases of blood-poisoning was absolutely full, and we were in some risk as regards the surgical cases in the other wards for women, since, had any patient after operation there been unlucky enough to develop erysipelas—and even in these days of most careful surgery such things will occasionally happen—we should have had to keep her where she was until a patient had gone out to leave an empty bed in the septic ward. Under these circumstances, having reached the utmost limit of our accommodation, what were we to do? Obviously it remained to call upon the Guardians to perform their duty to the sick poor to provide them with effective medical attendance, and this is where they fail to realise their own shortcomings, as is well pointed out in The Hospital newspaper (November 4th), a cutting from which I enclose; but it should be added in fairness to them that it is only since the passing of the London Government Act in 1899 that Guy's Hospital has been in the Southwark Union, and up to that time the people of Southwark had all the benefits and none of the inconveniences of a large London hospital in their midst. Hence a certain natural reluctance to modify their old arrangements though they no longer suit the changed conditions, and though Guy's, in addition to saving the Union large sums in outdoor medical relief, is now paying the Poor Rate upon a heavy assessment of many thousand pounds.

You may hardly be aware how complicated are the arrangements with the Poor Law officers of Southwark. Our doctors have no authority to send a patient direct to the Infirmary, however ill he may be. He has to go to the Relief Offices in the Borough Road, and these offices are closed between 12 noon and 3 p.m., as you may see from the enclosed notice. After 5 o'clock in the evening patients have to go to one or other workhouse—men to Mint Street, and women to Westmoreland Road, and it sounds incredible, but is true, that the Guardians have no complete arrangements at either place for cases, whether from Guy's or elsewhere, so ill as to require the comforts of a sick-ward, or so much injured that they cannot safely be taken three miles by ambulance to Dulwich. Their argument is that such cases ought never to be sent to them; our reply is that we never do send them until the limit of our available accommodation is reached, and that it is then their part to do what is obviously demanded, namely, to provide emergency beds and proper medical and nursing attendance centrally in their own area for cases that cannot be moved to Dulwich, and, for such cases as can, to have an efficient ambulance service at all hours of day and night. In this particular case, as you may notice, the house-surgeon so far short-circuited the regular procedure that, by favour of the medical superintendent, whom he consulted by telephone, he was enabled to send the patient direct to the Infirmary, instead of having her wait, either at the Relief Office or at Westmoreland Road.

In conclusion, I may add that a larger number of female beds for surgical and accident cases is greatly to be desired, and, by building new wards on the top of our present medical block, might, with sufficient support from the public, be provided, but in the meantime our beds are constantly worked to their full capacity, and the Guardians of Southwark seem unable to realise that, when the hospital can do no more, it remains for them to take their share of the burden.

(Signed) E. C. Perry.

